# High Flow and volume overload: The saga continues

**DOI:** 10.1590/2175-8239-JBN-2018-0002-0002

**Published:** 2018-06-25

**Authors:** Jennifer Cheng, Eric J. Costanzo, Tushar J. Vachharajani, Arif Asif

**Affiliations:** 1 Jersey Shore University Medical Center, Department of Medicine, Hackensack-Meridian School of Medicine at Seton Hall University, Neptune, New Jersey.; 2 University of North Carolina, Divisions of Nephrology, Chapel Hill, NC, EUA.; 3 Salisbury Salisbury Veterans Affairs Health Care System, NC, EUA.

In their article entitled, "Are high flow arteriovenous accesses associated with worse haemodialysis"^1^ the authors investigated a cohort of 304 hemodialysis patients. Of these, 48 patients demonstrated high flow. It is worth mentioning that high flow is defined as arteriovenous access flow exceeding or equal to 2.0 liters per minute. These investigators focused on evaluating two important components: 1) whether high flow fistulas were associated with reduced hemodialysis efficiency, and 2) whether high flow arteriovenous fistulas were associated with volume overload. The results disclosed that Kt/V was 1.99 ± 0.40 in fistulas with normal flow and 1.93 ± 0.35 in fistulas with high flow (non-significant). This finding is consistent with the notion that augmented access blood flow delivers adequate dialysis and does not lead to compromised Kt/V. While no difference was found in the adequacy of dialysis, Laranjinha and colleagues found high flow fistulas to be associated with volume overload. Their study defined volume overload categories as "dry weight" (absolute fluid overload below 1.1 liters), "volume overloaded" (absolute fluid overload above 1 liter) and "severe volume overload" (absolute fluid overload above 2.5 liters). Using these categories the investigators found that high flow fistulas were associated with severe volume overload.

The authors are to be commended for conducting such a study and initiate a dialogue about the impact of high flow fistulas on important dialysis parameters. Several investigators have provided the rationale for volume overload in patients with high flow fistulas^2^^-^ 3. Therefore, it is conceivable that high flow fistulas can be associated with volume overload. Their study^1^ revealed that severe volume overload was more prevalent in high flow fistulas (n = 4) when compared to normal flow fistulas (n = 6). Multivariate analysis demonstrated an odds ratio of 4.06 and a confidence interval of 1.01-16.39 with a *p* value of 0.056.

While the study was a bold attempt, a few elements raise concerns regarding the findings from both statistical and clinical standpoint. First of all, the sample size is extremely small (6 *versus* 4 patients). Second, the confidence interval is very large representing a small sample size. Third, the *p* value is slightly above significance (i.e. > 0.05). The evaluation of volume overload was performed by employing bioimpedance spectroscopy. From a clinical standpoint, volume overload is corrected by ultrafiltration. The authors do not provide information on the ultrafiltration rate. In support of the study, it is worth pointing out that both normal and high flow fistulas received the same duration of dialysis therapy (normal flow fistulas = 245 minutes, high flow fistulas = 246 minutes). Additionally, no information is available on the weight gain for the high flow fistulas. It is conceivable that the high flow fistulas had a higher weight gain due to a higher fluid intake.

**Figure 1:**
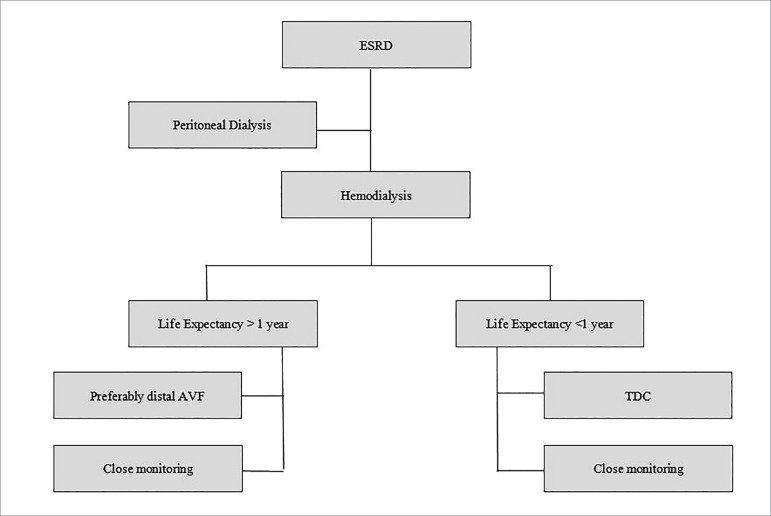
End stage renal disease (ESRD). Close monitoring of the access and life expectancy should be monitored. Modification to vascular access should be undertaken in the presence of a change in life expectancy. For instance, if life expectancy of a patient with catheter changes for the better, a distal (radial or ulnar artery based) arteriovenous fistula (AVF) should be considered.

The study, however, raised important concerns about high flow fistulas including their cardiovascular impact. A large sample size should explore the volume overload in high flow fistulas to conclusively establish their adverse impact on volume status. In the meantime, in an effort to avoid high flow fistulas, we suggest an opinion-based algorithm as shown in Figure 1. In this context, we prefer peritoneal dialysis (or renal transplant when available) as therapy of choice for end stage renal disease. However, if the patient chooses hemodialysis, we preferentially suggest the creation of a forearm radial artery or ulnar artery based fistula. At the same time, we suggest a tunneled hemodialysis catheter for patients with a life expectancy of less than a year (with ongoing evaluation of the life expectancy).
